# Determinants of quality of life and well-being in cognitively unimpaired older adults: a systematic review

**DOI:** 10.7717/peerj.12900

**Published:** 2022-02-10

**Authors:** María Dolores Frías-Luque, Abel Toledano-González

**Affiliations:** 1 Occupational Therapist, Fernán Núñez, Córdoba, Spain; 2 Neurological Disabilities Research Institute, Albacete, Spain; 3 Department of Psychology, Health Sciences Faculty, University of Castilla La Mancha, Talavera de la Reina, Toledo, Spain

**Keywords:** Factors, Emotional Intelligence, Quality of life, Healthy old people, Healthy aging, Well-being

## Abstract

**Objective:**

It is important to know the psychological variables that are related to quality of life and well-being in healthy elderly people. The main objective of the present review is to analyse which factors, through psychological variables, are determinant on the adaptive processes that acquire relevance in the last stage of life.

**Data sources:**

An electronic search was conducted in WOS, Science Direct, PsycARTICLES, Psychology Database and Psycinfo.

**Study selection:**

The search terms used were derived from the combination of the following search string: ((“Emotional Factors” OR “Emotional Effects”) AND (“Emotional Intelligence” OR “Emotional Regulation”) AND (“Quality of Life” OR “Personal Satisfaction”) AND (“Healthy Old People” OR “Healthy Old Adults”) AND (“Healthy Aging” OR “Successful Aging”)).

**Data extraction:**

11th April 2021.

**Data synthesis:**

In total, 13 articles were selected.

**Conclusions:**

The articles showed the importance of social support, proactive coping strategies (emotional regulation) and emotional intelligence as key factors in the elderly population for their positive influence on variables such as quality of life and well-being.

**Prospero ID: CRD42021224789:**

## Introduction

Quality of life is a construct composed of objective and subjective factors (*e.g*. social, environmental, health, *etc*.) (Fernández-Ballesteros, 2011b; Luque-Reca et al., 2015) of great importance in the 21st century. Therefore, in our study, this term has been taken into account from a multidimensional viewpoint to avoid having a reductionist concept of this construct (Fernández-Mayoralas et al., 2007).

In this sense, Cummins (1997) calls the concept of quality of life “a universal construct, both objectively and subjectively defined, in which the objective aspects would comprise culturally relevant measures of objective well-being and the subjective aspects would comprise satisfaction with the different dimensions weighted by their importance to the individual” (Cummins, 1997; Fernández & Pérez, 2005).

Specifically, we focus on “older adults” or “older people” throughout the study as the most used terminology (Fernández-Ballesteros, 2011a). More specifically, to those older people who are cognitively healthy, *i.e*., all those who do not have cognitive impairment or any mental illness that could condition the determinants related to quality of life by generating erroneous data.

Focusing on this specific group, it should be noted that, in the ageing process, a multitude of changes occur (decrease in autonomy, death of loved ones, *etc*.), and the adaptive value of emotions is relevant (Cosmides & Tooby, 2000). In this way, Emotional Intelligence (EI) acquires great importance, which is the set of skills used to perceive, express, understand and regulate both one’s own emotions and those of others, promoting personal and emotional growth (Mayer & Salovey, 1997).

Currently, two perspectives conceptualise EI. On the one hand, mixed models argue for EI as a multidimensional construct that includes cognitive skills, personality traits, and motivational and affective dispositions (Delhom et al., 2017). On the other hand, ability models consider EI to consist of a range of cognitive and emotional skills that link these two dimensions to understand and regulate emotions and/or produce feelings that facilitate thinking, with Mayer and Salovey’s skill model being the most influential (Delhom et al., 2017; Salovey & Mayer, 1990; Mayer & Salovey, 1997). According to the model proposed by Mayer and Salovey, EI is composed of four dimensions: the perception, appraisal and expression of emotion; the facilitation, assimilation or emotional use of thought; the understanding and analysis of emotions using emotional knowledge; and the reflective regulation or management of emotions to promote intellectual and emotional growth (Salovey & Mayer, 1990; Mayer & Salovey, 1997; Mayer, Caruso & Salovey, 2016). However, the articles reviewed do not focus on any particular model.

The study of EI during aging is an important topic to address (Labouvie-Vief et al., 2007; Marquez-González et al., 2004) as age is a modulating variable of this construct (Carstensen et al., 2011; Charles, 2011; Hay & Diehl, 2011) that provides adaptive strategies (Delhom et al., 2018). Thus, EI enables the older person to better adapt to the changes that occur in old age (Luque-Reca et al., 2015; Luque-Reca, Augusto-Landa & Pulido-Martos, 2016) and to improve coping skills (Chong, 2012) to promote successful and satisfying ageing (Chong, 2012; Luque-Reca, Augusto-Landa & Pulido-Martos, 2016), increasing levels of well-being (Rey & Extremera, 2012). This has led to several studies confirming the importance of EI in determining variables related to health (Austin, Saklofske & Egan, 2005; Martins, Ramalho & Morin, 2010), well-being (Austin, Saklofske & Egan, 2005; Zeidner, Matthews & Roberts, 2012) and quality of life of older people (Brackett et al., 2013; Ryff & Singer, 1996; Windle & Woods, 2004).

EI has also been found to be positively related to social support seeking (Delhom et al., 2018; Saklofske et al., 2007; Sarabia-Cobo et al., 2017). In addition, it has been shown that individuals with high EI experience fewer negative emotions immediately after a stressful stimulus occurs (Fernández-Berrocal, Extremera & Ramos, 2003) and, therefore, experience less stress and greater perceptions of health and well-being (Austin, Saklofske & Egan, 2005), which has a positive impact on their perceived quality of life (Ryff & Singer, 1996; Windle & Woods, 2004). In turn, these individuals have higher social support scores, indicating that they make better use of their coping strategies (Zeidner, Matthews & Roberts, 2012). This is reinforced by a study which shows that EI-based psychosocial interventions result in improved emotional skills, life satisfaction and coping in healthy older adults (Delhom, Satorres & Meléndez, 2020).

On the one hand, research indicates a positive and significant relationship between EI and psychological well-being. Psychological well-being is a multidimensional and complex construct associated with the achievement of personal goals and values that make us evolve as people and feel alive (Ryff, 1989). Specifically, EI is directly related to five of the six dimensions that make up Ryff’s scale (Self-Acceptance, Positive Relationships, Autonomy, Mastery of the Environment, Purpose in Life and Personal Growth) (Berrocal & Pacheco, 2004; Brackett & Mayer, 2003). These studies have subsequently been replicated, obtaining a moderate and significant relationship (Brackett et al., 2006; Fernández-Berrocal et al., 2006).

Moreover, emotional education has a positive influence on the psychological well-being of older people (Gross & John, 2003) and other studies show a significant relationship between personality variables and IQ scores (Berrocal & Pacheco, 2004; Mayer, Salovey & Caruso, 2008).

On the other hand, life satisfaction is based on attitudes and beliefs about one’s life (Diener, 2013) and is the cognitive component of subjective well-being (Diener et al., 2017). EI is necessary for the emotion management and mood and is a determinant of life satisfaction (Delhom, Satorres & Meléndez, 2020). Moreover, EI therefore has a significant, direct and positive relationship with the variables of life satisfaction and psychological well-being in older adults (Delhom et al., 2017), being determinants in the quality of life of these subjects.

The main objective of this review is to analyse the determinants that influence quality of life and well-being in cognitively healthy older adult people. These variables play a fundamental role in the development and maintenance of both the abilities and skills of the subjects, allowing a greater capacity for autonomy when carrying out activities of daily living. Thus, the improvement of the subject’s skills (psychological variables) could be directly related to a higher level of personal autonomy (Toledano-González, Labajos-Manzanares & Romero-Ayuso, 2019).

Therefore, we consider it essential to carry out such a review to evaluate the state of the research that has been carried out to date and to direct possible deficits in the right direction, with the psychological variables mentioned throughout the introduction and their direct influence on the well-being and quality-of-life of older people being of great importance.

This systematic review is necessary and important because, in old age, many changes occur such as the loss of loved ones, change of roles after retirement, *etc*., which mean that older people have to adapt to these events (Delhom et al., 2018). Therefore, it is important to know the main determinants that influence the well-being and quality of life of these people, paying special interest to the psychological variables that allow a better adaptation to the changes produced during this stage (Luque-Reca, Augusto-Landa & Pulido-Martos, 2016). In this way, knowledge of the main determinants resulting from this study and the subsequent work on them would allow us, in the near future, to design intervention programmes related to emotional education that could prevent mental illnesses such as depression or anxiety, shedding light on a worrying and current problem.

## Materials and Methods

The review was conducted according to the specific Cochrane Handbook for Systematic Reviews (https://handbook-5-1.cochrane.org/) and it was written following the PRISMA Declaration Guidelines. The investigators developed a protocol for this review, which was registered with PROSPERO (Prospective International Register of Systematic Reviews) before starting. (Registration number core CRD42021224789).

### Search strategy

To carry out the applicability and scope of this systematic review, a search strategy was carried out on the Web of Science (WOS), Science Direct, PsycArticles, Psychology Database and PsycINFO databases from 2010 to April 11th, 2021. The terms included in the search are shown in Table 1. This search string was applied to the fields Title + Summary + Keywords. The electronic search was completed with a review of the influence of psychological variables such as emotional intelligence (emotional factors) and quality of life in older adults of the included studies. The electronic search was complemented with a hand search of bibliographic references of the studies included. The search string was as follows “((*Emotional Factors OR Emotional Effects*) *AND* (*Emotional Intelligence OR Emotional Regulation*) *AND* (*Quality of Life OR Personal Satisfaction*) *AND* (*Healthy Old People OR Healthy Old Adults*) *AND* (*Healthy Aging OR Successful Aging*))” (See Table 1).

**Table 1:** Search criteria.

Limits	Studies
Chronology	Period 2010–2021
Language	English and Spanish
Categories	Psychology, Geriatric Gerontology and Gerontology

### Selection of studies

The selection of the articles in this systematic review was carried out through an exhaustive search of the aforementioned databases. Only articles that met the inclusion criteria listed below in Table 2 were included. Only studies related to older people and intervention on the variables in the table were considered for inclusion in this review.

**Table 2:** Criteria for inclusion and exclusion of studies.

Inclusion criteria	Exclusion criteria
Types of study: Descriptive studies and analytical studies (original research studies)	Studies that include only men or women solely
Item type: full text in English and Spanish	Studies that include elderly people with cognitive impairment or dementia
Variables: factors that influence emotional intelligence, emotional regulation or functioning of emotions and aspects of emotional intelligence and quality of life	Studies dealing with narrative revisions
Population: elderly people (more than 60 years)	Studies whose results are only preliminary

The selected articles directed towards psychological variables such as emotional regulation involved aspects intimately linked to quality of life and therefore with the possible autonomy of the participants, so that all of them should be considered as a set of variables that directly or indirectly affect the development of skills or abilities of older adults.

The minimum sample size of the studies was not restricted. Two independent reviewers (ATG and DFL) selected the studies and extracted the relevant data. Discrepancies were resolved by a third independent review (See Table 2).

### Data extraction and analysis

Both researchers (ATG and DFL) carried out the literature searches, and then reviewed the relevance of the titles found and abstracts according to the inclusion and exclusion criteria outlined in the process. Data extraction was performed by both investigators (ATG and DFL). Study quality was defined as whether the study results showed sufficient significance to result in symptom improvement (either through testing or treatment/intervention). A form was developed for the data extraction phase which included the following data: article title, year, authors, study type, sample size, follow-up, participant characteristics, description of the experimental and control intervention, main results and conclusions.

The objective measures of effect used to assess the effect of the intervention related to the influence of psychological variables in healthy older people on the variables of emotional regulation, emotional intelligence, emotions, and quality of life. Emotional regulation was analyzed from different perspectives using the structured interview with the aim of verifying its possible influence on quality of life in the target population.

## Results

A total of 256 studies were identified. Of these, eight studies were duplicates and were automatically discaraded. Once the duplicates were removed, 248 manuscripts were reviewed for title and abstract, and 188 were excluded.

From the 60 studies that remained in the final full-text eligibility phase, 47 articles were eliminated for the following criteria: 15 because they did not meet the adult or older adult theme, seven had only one sample type in terms of gender, 18 had a sample of people under 60 years, 4 included people with advanced cognitive impairment, two were focused on narrative critique and one pertained to an evaluation project or protocol.

Finally, the studies included in the final phase for inclusion in this systematic review were 13 articles. All of them met the characteristics of the objective set at the beginning and were proposed to analyze their internal quality and verify it through an evaluation table.

The scarce scientific evidence on the analysis of the main determinants that influence the well-being and quality of life of older adults without cognitive impairment, mainly taking into account psychological variables, leads us to consider the need to develop this systematic review in order to provide useful and relevant information on this subject.

**Figure 1:**
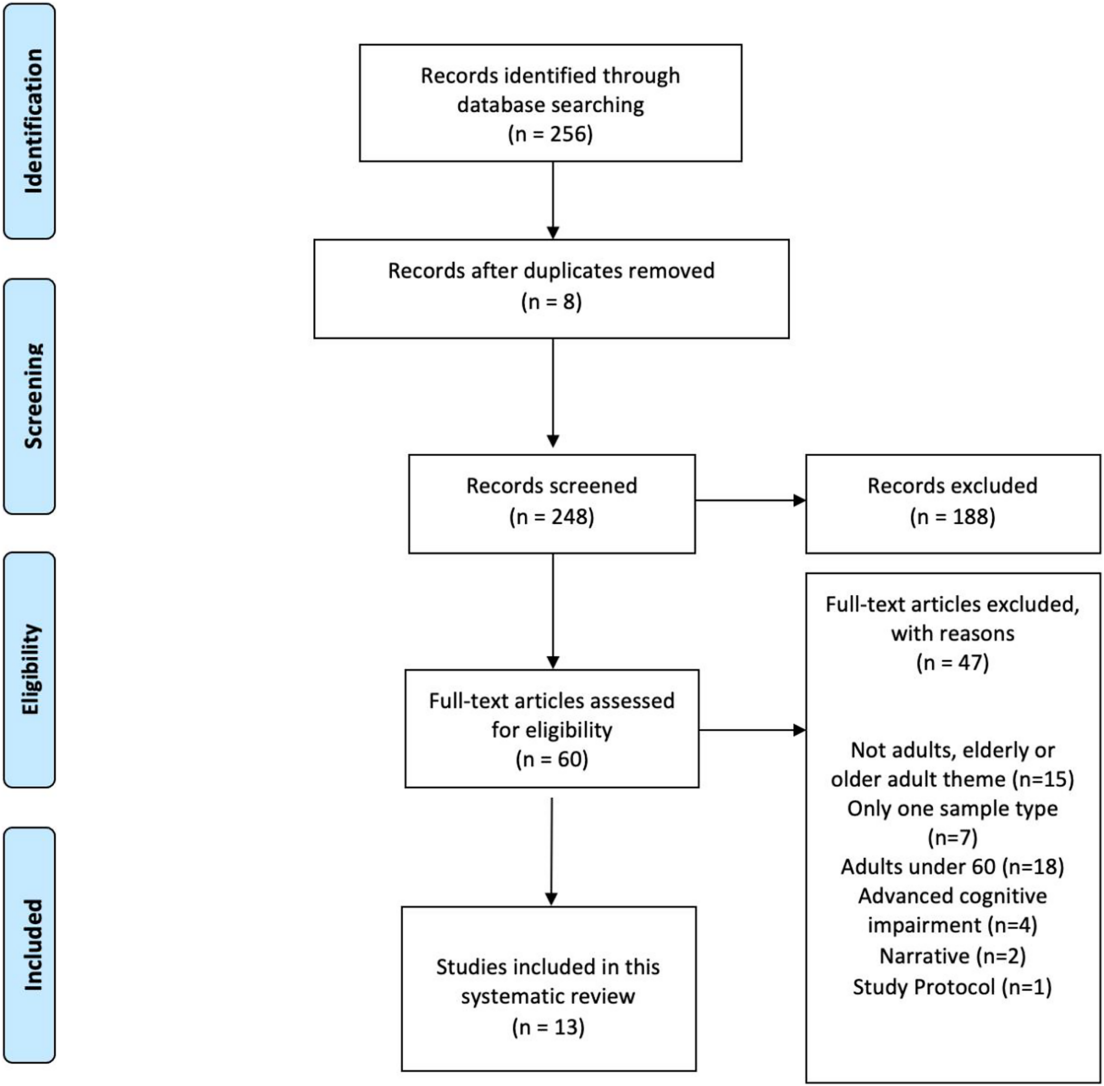
Flowchart of the PRISMA criteria. Source: own elaboration.

For this reason, a small number of studies make up our review after applying the inclusion and exclusion criteria (See Fig. 1). Most of them deal with the relationship between psychological variables and how these can positively influence coping strategies to adapt to the changes that arise during ageing. These strategies have a positive impact on life satisfaction, subjective well-being and quality of life of older people.

### Characteristics of the studies included

Based on the total number of 13 studies included in this review published in the last 10 years, studies related to emotional regulation and quality of life in older adults were taken into account to measure the quality of the results (See Table 3).

**Table 3:** Criteria list of assessing the methodological quality of studies.

A	Socio-demographic and medical data is described (*e.g*., age, race, employment status, educational status, stage at diagnosis *etc*.).
B	Inclusion and/or exclusion criteria are formulated.
C	The process of data collection is described (*e.g*., interview or self-report *etc*.).
D	The type of treatment is described.
E	The results are compared between two groups or more (*e.g*., healthy population, groups with different treatment or age, comparison with time at diagnosis *etc*.).
F	Mean or median and range or standard deviation of time since diagnosis or treatment is given.
G	Participation and response rates for patient groups have to be described and have to be more than 75%.
H	Information is presented about patient/disease characteristics of responders and non-responders or if there is no selective response.
I	A standardized or valid quality of life or emotional experience questionnaire is used.
J	Results are not only described for quality of life but also for the physical, psychological and social domain.
K	Mean, median, standard deviations or percentages are reported for the most important outcome measures.
L	An attempt is made to find a set of determinants with the highest prognostic value.
M	Patient signed an informed consent form before study participation.
N	The degree of selection of the patient sample is described.

To carry out the analysis of the quality of the studies, we used a list of methodological criteria related to the subject through the following items:

The quality of the included studies is shown in the table below. The range of scores remains around 8–12 points. The average quality of the quality collected is 9.4. There is a high quality of the results in all aspects collected. Methodological shortcomings concerned mainly the response rate and the lack of information on characteristics of non-responders (See Table 4).

**Table 4:** Overview of studies on quality of life and emotional intelligence among older adults.

Study	Study Quality	Participants	Country	Age (yrs)	Design	Instruments	Results	General Conclusions
Ardelt & Jeste (2018)	12/14	994 61–99 Years 48% Female 52% Male	USA	x̄ = 77.3 SD = 12.2	Randomized cohorts’ study by intervention type	SF-36 Life Events Scale 3D-WS SWB CES-D	The bivariate correlations show indicators associated with the well-being of the 3 dimensions of wisdom (reflective *p* < 0.05, compassionate *p* < 0.05 and cognitive *p* < 0.05). Regarding three-dimensional wisdom and well-being, it was positively related to all SWB variables *p* < 0.0001. On the other hand, the interaction effect between composite wisdom and adverse life events on SWB was positive and significant with main effects on well-being (ΔX^2^ = 14.2, Δ df = 1, *p* < 0.001).	Subjective well-being, quality of life and adverse experience were positively associated within the reflective wisdom dimension. Cognitive wisdom has high associations with an orientation towards personal growth, autonomy, and openness to new experiences. These 3 dimensions enhance aspects such as emotional regulation, personal growth orientation, purpose in life, positive relationships with others and ethical attitudes. The greater number of psychological resources on the phenomena and events that occur around them allow them to preserve a greater sense of well-being when faced with critical moments or great difficulties.
Brett et al. (2012)	10/14	Study 1: 550 43% Male 57% Female Study 2: 1091 50.2% Male 49.8% Female	Scotland	n/d	Cohort’s study	WHOQOL-BREF IPIP HADS JAMAR	Significant associations were found between the 5 quality of life measures and the independent variables. Correlations ranged from −0.092 to −0.536 in study 1 and −0.060 to −0.565 in study 2, showing higher correlations between quality-of-life measures and constructs of depression, anxiety, emotional stability and quality of life. Quality of life factors and the environmental and social domains correlated significantly with most of the independent variables, however, the psychological and physical domains had lower power. Regression analyses explained 46.4%/44.5% of the variance in the general quality of life factor and 19.76/18.7% of the variance in the social domain. The presence of fewer depressive symptoms contributed to higher quality of life scores. Emotional stability played an important role in addition to extraversion in both psychological and physical domains.	Personality traits, especially emotional stability, and minor depressive symptoms, have a significant influence on the self-formed curriculum vitae in the development of one’s own old age. The strong relationship between mood and quality of life suggests that interventions aimed at improving the individual’s functional status can improve his or her mental health to increase his or her subjective well-being. Moreover, high correlations between sociodemographic variables, the aging process and the quality of life of the participants allow highlighting the interesting general factor of quality of life in the HADS models of depression, emotional stability, living alone and awareness. Taking into account the personality traits of individuals allow to detect how those participants with a low level of emotional stability may be more prone to exaggerate physical symptoms and experience negative emotions in response to difficult circumstances, consequently a lower quality of life.
Dockendorff (2014)	8/14	36 65–99 Years 44% Male 56% Female	Chile	n/d	Quantitative and qualitative study.	Functional Assessment Scale of Seniors Mini Mental Exam	Quantitative results corroborated the qualitative results through the associations of high levels of SWL with the item accommodation and low levels of SWL with the item submission. Spearman’s Rho correlations (0.01 significance) allow for relationships between coping skills and types of loss, the most significant being isolation (−0.517; *p* = 0.001), accommodation (0.379; *p* = 0.023), escape (−0.347; *p* = 0.038).and submission (−0.290; *p* = 0.086). Accommodation has a positive relationship with SWB while isolation, submission, escalation and self-reliance are negatively correlated across non-adaptive coping strategies.	This research allows us to analyze the possibilities of promoting well-being in the elderly through the design of prevention and intervention strategies. The results show that a high level of subjective well-being is associated with severe losses, such as progressive blindness, and low levels are associated with milder situations, such as moving house. The forms of coping in the qualitative analysis showed a higher correlation with SWB reinforcing the idea that healthy coping strategies are effective psychological resources in old age.
Dumitrache, Rubio & Cordon-Pozo (2019)	9/14	406 65–99 Years 38% Male 62% Female	Spain	x̄ = 74.88 SD = 6.75	Cross-Sectional Study	Satisfaction with Life Scale LOT-R MOS	The results shown through the structural model analysis allowed explaining 47.4% of the variance regarding life satisfaction, being 7 of the 13 hypothesized associations significant at least at 95% (age β = 0.129 y T = 3.470; optimism β = 0.173 y T = 3.811; extraversion β = 0.191 y T = 4.467; social support β = 0.107 y T = 2.009; satisfaction with family β = 0.153 y T = 3.044; time with family β = 0.088 y T = 2.676; and satisfaction with financial resources β = 0.162 y T = 3.565). On the other hand, 5 associations were marginally significant (gender β = −0.069 and T = 1.811; size of social network β = 0.058 and T = 1.537; satisfaction with friends β = 0.079 and T = 1.741; functional limitations β = −0.091 and T = 1.903 and subjective health β = 0.075 and T = 1.526).	Social relations and personality, especially optimism, were strongly related to satisfaction with life, while health status and sociodemographic characteristics were modestly associated with satisfaction with life. It shows advances in knowledge about the importance of psychosocial resources for life satisfaction and successful ageing by identifying specific resources such as optimism and social relationships.
Elran-Barak et al. (2018)	10/14	481 60–93 Years 31% Male 69% Female	Israel	x̄ = 77.4 SD = 6.7	Cross-Sectional Correlational Study	CES-D Self-Widening Scale Self-Assessed Scale	Between-group comparisons on subjective means of well-being showed significant differences in self-rated health and depressive symptoms, but not in life evaluation. Holocaust survivors were significantly more likely to mention responses about “Maintaining good health” (39%), compared to post-war (27.9%) and pre-war (21.6%) immigrants (*p* = 0.004). Logistic regression analyses on the “maintain good health” and “enjoy” items showed that Holocaust survivors were 2.2 times more likely to use the “maintain good health” category relative to pre-war immigrants (*p* = 0.09). On the other hand, pre-war immigrants were 1.9 times more likely to use the category “enjoyment” (*p* = 0.059).	People ageing in Israel who have suffered childhood or early adulthood trauma use proactive (health) and cognitive (acceptance of the present) coping methods, regardless of their declared history during the war. The early life experiences of 85% of the study’s participants due to the conflict situation have enabled them to adopt a different philosophy of life that could determine their priorities in old age.
Frías (2014)	9/14	89 65–85 Years 31% Male 69% Female	USA	x̄ = 64.13 SD = 2.83	Cross-Sectional Study	PHQ-9 MCQ SF-36v2 ERQ MAAS	Emotional regulation strategy was a clear predictor of memory compensation (internal mnemonic strategies R2 = 0.26). Bream mindfulness was related to 5 MCQ strategy scales (external R2 = −0.30, internal ΔR^2^ = 0.26 The sergeant’s mindfulness was related to 5 MCQ strategy scales (external ΔR^2^ = −0.30, internal ΔR^2^ = −0.36, recruitment ΔR^2^ = −0.36, time ΔR^2^ = −0.41 and effort ΔR^2^ = −0.42). Emotion regulation or attention to traits accounts for 10–14% of the variance. According to the moderating effects of age, physical health effects influenced internal MCQ and MCQ Effort. Better physical health was related to more frequent use of internal mnemonic strategies β = 0.17, *p* < 0.05 and effort investment β = 0.26, *p* < 0.05 Age moderated effects on mental health in MCQ Recruitment β = −0.45, *p* < 0.05 y MCQ Effort β = −0.26, *p* < 0.05. Age interactions accounted for 10% of the variance.	HRQoL, coping strategies to regulate emotions and trait mindfulness are additional contexts that determine the degree of engagement in daily memory compensation. Individual-level resources are linked to daily memory compensation in older adults. Poor mental and physical health predicts a higher frequency of use of compensatory strategies. On the other hand, cognitive reappraisal as an emotional regulation strategy predicted the use of internal mnemonic strategies, specifically high attention span being associated with lower use of compensatory strategies.
Gamrowska & Steuden (2014)	8/14	88 60–85 Years 14% Male 86% Female	Poland	x̄ = 69.5 SD = 6.74	Correlational Study	PCI CASP-19	Pearson’s correlation analysis of the relationship between the variables proactive coping and quality of life (*p* = 0.53). Associations were shown at a moderate level, being positive in all 3 dimensions of quality of life with emphasis on control *p* = 0.49, pleasure *p* = 0.49 and overall quality of life outcome *p* = 0.40. Participants who scored between 80 and 123 (*M* = 110) were classified as low proactive coping (NPR) and those who scored between 148 and 197 (*M* = 162.5) were classified as high coping (WPR). Significant differences between the two groups were concentrated in the General Quality of Life items (*p* = 0.003) and the dimensions of Control (*p* = 0.000) and Pleasure (*p* = 0.000).	Contributing factors are autonomous goal setting, initiative, perseverance and the perception of events in terms of opportunities for development and self-improvement, with the maintenance of activity and well-being being important in counteracting possible social stereotypes of old age and risk factors for exclusion. Aspects such as quality of life, the degree of satisfaction of the individual’s own needs enable proactive corrective strategies to be established.
Huxhold, Fiori & Windsor (2013)	8/14	2034 +65 Years 52% Male 48% Female	Germany	x̄ = 73.72	Cohorts Study	Network Variable Questionnaire Life Satisfaction Scale	Analysis of the associations between health and well-being domains, achieving a satisfactory overall model fit X^2^(41) = 75.58; CFI = 0.99; RMSEA = 0.02; SRMR = 0.02, with the fit for the health model being X^2^(70) = 248.94; CFI = 0 .98; RMSEA = 0.04; SRMR = 0.04. With respect to the social network domain, social networks are associated with factor levels and factor changes. Age was negatively associated with both the level of network structure T1, β = −0.16; X^2^(1) = 45.52; *p* = 0.000; and with the level of activity T1, β = −0.16; X2(1) = 160.15; *p* = 0. 000; On the other hand age was also associated with the level of emotional support T1, β = −0.08; X^2^(1) = 8.15; *p* = 0.004; Emotional changes were predicted by inter-individual differences in network structure β = 0.32; X^2^(1) = 34.53; *p* = 0.000; On the other hand, social factors affecting health and well-being were found, according to which a higher level of potential emotional support would lead to a decrease in negative affect. Higher levels of activity engagement were also found with greater changes in life satisfaction β = 0.11; X^2^(1) = 6.31; *p* = 0.012; This leads us to believe that increased social engagement is a predictor of life satisfaction and higher levels of emotional support.	During ageing, changes in social engagement were associated with changes in the development of interactive processes (individual resources and outcomes). These in turn modify other factors such as life satisfaction, personal autonomy, functional health, and objective health. They all provide different items to optimize various facets of successful ageing and provide insight into the dynamic interaction between social networks and adaptive resources.
Leanos et al. (2020)	12/14	Study 1: N = 15 60–74 Years 6 Male 9 Female Study 2: N = 27 60–86 Years 9 Male 18 Female	USA	x̄ 1 = 66.33 x̄ 2 = 69.44	Clinical Trial	NIH Examiner RAVLT IADL	Study 1: Mean composite cognitive scores were 0.225 units higher than pre-test scores (*p* = 0.014) although no significant differences were found. In cognitive control, significant gender differences were found (*p* = 0.013), being higher in the post-test for males compared to the pre-test (*p* < 0.001). Regarding working memory, the pre-test results were different (*p* = 0.014) but without differences between groups. The mean score increased by 0.360 units. MMSE scores became a significant predictor (*p* = 0.097) for working memory. In the episodic memory section the interaction between time and group was significant (*p* = 0.012), being higher for the control group (1.588 units) than for the experimental group (1.55 units). Regarding everyday problems, EFA scores in the intervention group increased by 12% reaching 86.19% in the post-test (M = 12%; SD = 12%; Range = −1% to 31%). While in the control group the scores decreased from 81.11% in the pre-test to 78.62% in the post-test M(−2%; SD = 12%; Range = −22% to 16%). Study 2: In cognitive control scores, a significant increase was detected in both sexes (*p* = 0.043) although without significant differences with respect to the control group. For working memory, scores increased with respect to baseline (*p* = 0.001) and post-test (*p* = 0.033). A significant increase of 0.034 units was detected between pre and post working memory scores (*p* = 0.004). In the episodic memory section there was a significant increase of 1,548 units from baseline (*p* < 0.001) and from post-test of 2,622 units (*p* < 0.001). MMSE scores were a significant predictor (*p* = 0.011) of RAVLT score. On the other hand, the gender variable was particularly significant in women compared to men (*p* = 0.041).	Exposure of older adults to a novel and intense learning environment allows for the analysis of learning abilities in psychological aspects where determining factors come into play with respect to the impact on long-term functional independence, real-world learning skills, the ability to adapt to a dynamic environment and the ability to successfully complete basic daily tasks. Measures related to the willingness to acquire skills, experience in performing cognitive and functional skills allow for improved EFA (IADL) scores, with the aim of remaining active and enabling successful ageing without altering integral factors related to cognitive growth.
Martin et al. (2015)	9/14	1006 60–99 Years 51% Male 49% Female	USA	x̄ = 74.3	Cohorts Study	PHQ 9 SF-36 SWLS TIC-M CD-RISC LOT-R PSS	Significant differences were found between age, marital status and education level groups. The O–O group had higher SPSA scores for self-assessment of successful ageing (*p* = 0.003), higher life satisfaction (*p* = 0.001), lower physical functioning (*p* = 0.001), higher mental functioning (*p* = 0.001), higher subjective cognitive concerns and higher objective cognitive impairment (some of them resulting from age-related decline). Lower SPSA scores were found to be associated with age (*p* = 0.017), female gender (*p* = 0.032), marital status (*p* = 0.455) and mental (*p* < 0.001) and physical functioning (*p* < 0.001). A higher SPSA score was significantly associated with higher physical functioning. Regarding perceived stress (*p* = 0.043) and depressive symptoms (*p* = 0.124), the interaction with age group were small regression coefficients, indicating greater weakness of the explanatory variables with SPSA.	It is important to know, due to the high growth of the elderly population, which are the determining factors from the psychological variables that are associated with aspects of active and satisfactory self-perceived aging as an essential part of successful aging. On the other hand, given the difficulty presented by the constructs associated with psychological variables, there are conditioning factors that can alter these factors and that should be considered, such as the results of sociodemographic variables, such as educational level, and the experience of the older adult, as an aspect aimed at adapting the underlying interventions that promote or allow successful aging to be achieved.
Meeks et al. (2012)	9/14	815 +60 Years 38% Male 62% Female	USA	x̄ = 73.5 SD = 9.85	Prospective Study	Bradburn Affect Balance Scale Philadelphia Geriatric Center and Negative Affect Rating Scale Rosenberg Self-Esteem Scale Life Satisfaction Index QOL-AD	The results showed a correlation between well-being, self-esteem and social support scores (General Well-being Scale) comparing both groups among which a significant difference was marked between the well-being groups T(621) = 6.64; *p* < 0.001. On the one hand, 9% had higher well-being with a positive relationship between the level of mental health and years of education received, arguing that proportionally lower levels of perceived well-being are caused by a lower level of education. On the other hand, 20% of the participants were found to have very high levels of. Well-being T(621) = 12.21; and T(184) = 9.25 (Levene’s test *F* = 4.67; *p* = 0.03). The relationship between a high level of general well-being and life satisfaction was 51%, with this group showing high scores on all target variables. The *F* values ranged from 6.85 to 409.23 and were significant except for the lowest (*p* < 0.001). Finally, associating the affect scale with well-being, statistically significant differences were found between the groups T(39) = −5.33; *p* < 0.001. This relationship implies high well-being for those older people living in the community because of the social support they receive. High well-being implied higher activity in pleasurable events (higher frequency). In this section there were no major differences with respect to the participant’s physical condition.	The context can influence psychological aspects of the elderly, such as the capacity to regulate positive affect, which is greater in people who continue to live in the community, and how people with a good quality of life can determine a better emotional adaptation to fewer ideal circumstances (acceptance of the changes that occur with the passage of time). Therefore, changes in positive and negative affect determine intraindividual changes accompanied by well-being. Such direct belonging implies working on aspects such as resilience or helping older adults to improve their adaptation.
Moreno et al. (2014)	8/14	154 65–96 Years 50% Male 50% Female	Spain	x̄ = 77.44	Cross-Sectional Study	HS Scale Balance Scale of Affection GHQ-28 SF-12 SES Scale Social and Emotional Support Scale Scale of Stressful	The results revealed significant differences for all variables among participants in relation to average coping and stress *p* = 0.068, significant differences among highly adapted elders according to psychosocial variables *p* < 0.01 except for SF mental health *p* < 0.05. On the one hand, differences were found between the elderly groups due to age (*F* = 16.588, *p* = 0.000); these being among the more disabled elderly. On the other hand, the elderly who were more successful in the questionnaires showed higher levels of positive affect (*p* = 0.000), affective balance (*p* = 0.007) and happiness (*p* = 0.000) than the average. The variable age was closely related to life satisfaction (*F* = 5.546; *p* = 0.020), as well as group membership to life satisfaction by eliminating the age effect (*F* = 19.240; *p* = 0.000). Another important aspect related was the fact of "having children" with affective balance (*F* = 5.465; *p* = 0.021), being higher in those older people who had children than in those who did not (*F* = 2.338; *p* = 0.021).	The analysis of the variables in this study have allowed us to understand how older adults who had a better adaptation presented greater success in their abilities due to high levels of happiness, life satisfaction and affective balance. In addition, we analyzed the relationship between the different dimensions of SWB and the possibilities that are marked from the psychosocial profiles that determine different trajectories of aging. The results suggest that older people maintain a greater sense of well-being in the face of risks, losses or declines that occur during their trajectory, not being associated with negative outcomes but with the capacity or use of psychosocial resources for successful or positive aging.
Wurm et al. (2013)	10/14	309 65–85 Years 58% Male 42% Female	Germany	x̄ = 73.27 SD=5.1	Case-Control Study	Life Satisfaction Program Scale SF-36 Self-Qualified Health SOC	The results show predictor values between physical functioning, perceived health and life satisfaction, with the post-test being a predictor of life satisfaction (β = −0.16, SE = 0.09; *p* < 0.05), but not a predictor for the physical functioning variable (β = −1.03; SE = 2.44; *p* > 0.05). On the one hand, perception of ageing was associated with selection, optimisation and compensation as a strategy in the post-test which was significant (β = −0.12; SE = 0.06; *p* < 0.05). The lower the self-perception of ageing, the higher the selection, optimisation and compensation strategies were. A self-perception of ageing was associated in the post-test with a higher use of compensatory strategies (β = −0.41; SE = 0.20; *p* < 0.05), having an indirect effect on physical functioning. On the other hand, life satisfaction was associated with compensatory strategies at both measurement and post-test (β = 0.21; SE = 0.06; *p* < 0.001) as well as with perception of ageing (β = 0.25; SE = 0.14; 95% [0.045–0.651]. The lower the perception of ageing, the lower the satisfaction with life and therefore the lower the use of compensatory strategies.	The analysis of the effect of negative self-perception, quality of life and psychological variables during ageing and the use of self-regulation strategies in the face of the events that occur during the process play a fundamental role in the individual. Importance of health and life satisfaction in the recovery of the use of compensatory strategies to minimise the deficits that may arise at a cognitive level.

**Notes:**

The second column represent “study quality”. Each item of a selected study, which met our criteria, was assigned one point. If an item did not meet our criteria, was described insufficiently, or not at all, zero points were assigned. The highest possible score was thus 14. Studies scoring 75% or more of the maximum attainable score (ie more than 10 points) were arbitrarily considered to be of “high quality”. Studies scoring between 50% and 75% were rated as moderate quality. Studies scoring lower than 50% were considered as low quality.

SF-36 (Short Form Healthy Survey 36), 3D-WS (Three Dimensional Wisdom Scale), SWB (Subjective Well-Being Scale), CES-D (Center of Epidemiological Studies-Depression), WHOQOL-BREF (World Health Organization Quality of Life Questionnaire), IPIP (International Personality Item Pool), HADS (Hospital Anxiety and Depression Scale), JAMAR (North Coast Hydraulic Hand Dynamometer), LOT-R (Life Orientation Test-Revised), MOS (Mean Opinion Score), PHQ-9 (Patient Health Questionnaire-9), MCQ (Multiple Choice Questions), ERQ (Emotion Regulation Questionnaire), MAAS (Mindfulness Attention Awareness Scale), PCI (Pavement Condition Index), CASP-19 (Control, Autonomy, Self-Realization and Pleasure 19), NIH Examiner (Executive Abilities: Measures and Instruments for Neurobehavioral Evaluation and Research), RAVLT (Rey’s Auditory Verbal Learning Test), IADL (Instrumental Activities of Daily Living Scale), SWLS Scale (Satisfaction With Life Scale), TIC-M (Telephone Interview for Cognitive Status), CD-RISC (Connor-Davidson Resilience Scale), PSS (Perceived Stress Scale), QOL-AD (Quality of Life Alzheimer’s Disease), GHQ-28 (Goldberg General Health Questionnaire), SF-12 (Short Form Healthy Survey 12), SES (Socio-Economic Status), SOC (Sense of Coherence).

Our results indicate that coping strategies are important for emotion regulation, namely proactive strategies or the perception of events in terms of development and coping. This is related to life satisfaction, subjective well-being and quality of life of the cognitively healthy.

We found that socio-demographic characteristics, social engagement, social relationships and personality are strongly related to life satisfaction, being factors that influence EI. In addition, adverse experience, personality traits and emotional regulation strategies are factors that have an impact on subjective well-being.

In general terms, adverse experience, socio-demographic variables, social engagement and context are factors that influence the quality of life of the older person, with happiness, life satisfaction and affective balance being favourable factors for adaptation to ageing. More specifically, we can point out that the findings found in the studies included in our systematic review can be grouped according to the different variables taken into account for the development of this study, with this grouping being as follows:

**–Coping strategies and emotional regulation:**
  Selection, optimisation and compensation (SOC) strategies were mainly used by older adults when a serious health event occurred, although their use was lower if the event was perceived and associated with physical losses (Wurm et al., 2013). However, another study shows that compensatory strategies were mainly used in older adults with poorer physical and mental health (Frías, 2014).
  Some studies support the proactivity model of ageing by highlighting coping strategies (except avoidance coping) with a positive relationship with subjective health indicators, life satisfaction, optimism and quality of life (Gamrowska & Steuden, 2014) and it was confirmed that older people use proactive and cognitive coping methods as they age (Elran-Barak et al., 2018).
  Therefore, healthy coping strategies were found to be effective psychological resources that help to understand the paradox of well-being in old age and are essential for maintaining high levels of subjective well-being in old age (Dockendorff, 2014). The use of the strategy depends on the severity of the health event that occurred and the consequences it has for indicators of successful ageing (Wurm et al., 2013).
**–Satisfaction with life**
  Social relationships and personality, specifically optimism, were strongly related to life satisfaction and both were considered psychosocial variables influencing emotional intelligence (Dumitrache, Rubio & Cordon-Pozo, 2019). In turn, changes in social engagement were also associated with changes in life satisfaction (Huxhold, Fiori & Windsor, 2013). Thus, social relationships, personality and changes in social engagement had a direct relationship with life satisfaction and were considered influential factors in emotional intelligence and had an impact on the quality of life of the people in our study.
**–Subjective well-being:**
  In terms of context, one study showed that institutionalised older adults were happier and better adjusted (Moreno et al., 2014). However, another study showed that context does not influence well-being, as both institutionalised and non-institutionalised older adults had high levels of well-being (Meeks et al., 2012). Therefore, there is no clear evidence regarding the relevance of context as a factor influencing emotional intelligence and its impact on the quality of life of the mentally healthy older person.
  Furthermore, changes in social engagement were related to changes in subjective health (Huxhold, Fiori & Windsor, 2013). On the one hand, education was important as older people with a high level of education showed higher levels of self-perceived successful ageing associations (Martin et al., 2015). On the other hand, wisdom as a reflective dimension had a significant association with subjective well-being and buffered the negative association between adverse life events (Ardelt & Jeste, 2018).
  However, one study showed high levels of subjective well-being in the face of losses that could be considered severe, such as progressive blindness (Dockendorff, 2014).
**–Quality of life:**
  Changes in social engagement are also closely related to changes in functional health (Huxhold, Fiori & Windsor, 2013) strongly related to quality of life. Furthermore, simultaneous learning of multiple skills is feasible and potentially beneficial for healthy older adults (Leanos et al., 2020).

In addition, socio-demographic variables such as gender, age, social class, years of education and mainly social support were relevant to the older person’s quality of life (Brett et al., 2012). Therefore, social engagement and simultaneous learning of multiple skills are recognised as factors that influence EI and positively impact the quality of life of the cognitively healthy older person.

Finally, we would like to emphasise that all factors that influence EI and have an impact on life satisfaction and/or subjective well-being discovered through this systematic research contribute to the quality of life of the older person as they are essential components of quality of life.

## Discussion

The results gathered during the present systematic review acquire a relevant importance according to the indications shown by the resulting articles. Psychological variables affect aspects related to social support and personality, which in turn influence others such as the emotional intelligence of the older adults and can condition either positively or negatively life satisfaction and, consequently, the perceived quality of life or well-being of older adults, as can be found in the articles mentioned (Delhom et al., 2017; Wilson & Saklofske, 2018).

Another important aspect that allows higher emotional intelligence, life satisfaction or subjective well-being scores to be shown is through perceived social support. In the research by Rey, Extremera & Sánchez-Álvarez (2019), relationships between the scores of these variables are observed, with participants reporting that a higher level of perceived social support from people close to them translated into higher scores for quality of life and well-being. This implies the fundamental role that emotional intelligence may play in the perceived well-being and quality of life of older people, as do the rest of the articles in our review (Rey, Extremera & Sánchez-Álvarez, 2019).

On the other hand, it seems reasonable that the well-being perceived through the skills that make up emotional intelligence can play a predictive role on the quality of life and well-being perceived by the same person, allowing to enhance the ability not only to understand but also to manage those life events that may be perceived as negative or stressful (Sánchez-Álvarez, Extremera & Fernández-Berrocal, 2016). Furthermore, these aspects are also supported by other research that analyses similar aspects or situations in which they are perceived as potential or conditioning factors on the influence of these psychological variables (Melero Aguilar & Fleitas Ruíz, 2015; Ramírez Pérez & Lee Maturana, 2012).

In terms of personality, the scientific literature refers to the relationship between different personality traits (emotions, ways of thinking and aspects related to the individual’s behaviour) which can also be considered as psychological strengths, through a buffering effect on the associations between life circumstances and quality of life (Ramirez, Ortega & Martos, 2015). These previously described traits allow providing information about social support and therefore their emotionally intelligent behaviours, allowing those with high emotional intelligence to have greater access to social support and therefore greater perceived well-being (Koydemir et al., 2013).

In line with this view, stressful life events, such as the loss of a loved one or a high-stress event, can negatively affect the emotional behaviour shown by individuals either at the initial phase, during or after the stressful event (Kraaij, Arensman & Spinhoven, 2002), having a high impact on cognitive, social and physical aspects of older people (Kok et al., 2017). Thus, there is a coping style that focuses on how emotions allow a response to the changes that are generated in the face of coping resources learned throughout life or the person’s own experience based on previous experiences (Carver & Connor-Smith, 2010). Specifically, ageing is associated with a greater capacity for emotional regulation in context, generally due to one’s own experience, allowing for a greater response based on previously experienced events (Carstensen, 2006).

On the one hand, there is a positive relationship between proactive coping strategies (with the exception of avoidance behaviour) and quality of life in older people. This relationship is associated with subjective indicators of health, life satisfaction and optimism (Gamrowska & Steuden, 2014). All these changes that occur during the ageing period lead to changes in emotional capacities, well-being and sources of social support, allowing for changes in the way cognitive aspects of emotional stimuli, emotional competence and subjective well-being are processed (Scheibe & Carstensen, 2010; Urry & Gross, 2010). All of these data from the aforementioned articles discuss the modifications that take place through proactive coping and how this can influence the positive outcome of adaptation to the ageing process (Almássy et al., 2014; Greenglass & Fiksenbaum, 2009).

One of the studies included in the review refers to how concurrent learning is potentially beneficial for healthy older adults (Leanos et al., 2020). Thus, emotional experience allows them to adapt to special or high-stress situations such as the pandemic through COVID-19 in the entire world population (Malone et al., 2020) and other problems associated with skills that allow the control of emotions, or avoidance behaviours about situations that generate great discomfort, choosing those situations with which they obtain emotionally comfortable or positive experiences (Arias, 2013).

Previous scientific literature also reflects the importance of potential or very important aspects such as emotional education in older people when faced with the appearance of any type of situation or state of health, for example, an illness, with the aim of reducing or minimising the impact that this may have on the person’s quality of life and therefore improving the level of perceived well-being (Ortega Navas, 2010).

Specifically, the negative emotions that can be generated from adverse situations can make older people more vulnerable to diseases that affect a high rate of the variables analysed, such as Alzheimer’s disease, heart disease and even cancer. These types of emotions that are secondary to the aforementioned pathologies can have a high probability of influencing the appearance, development and maintenance of physical and mental illnesses (Rodríguez et al., 2009), negatively affecting quality of life, subjective well-being, autonomy and personal independence (Ortega Navas, 2010; Rodríguez et al., 2009; Toledano-González, Labajos-Manzanares & Romero-Ayuso, 2018). However, if anything can increase this situation, it is positive emotions, which in some cases can even boost the immune system and quality of life, leading to recovery from certain diseases and are beneficial to health (Ortega Navas, 2010). They also facilitate creative problem solving, strengthen in the face of adversity (Lyubomirsky, King & Diener, 2005) and protect against depression (Fredrickson, 1998). Positive emotions are therefore a pillar that leads to well-being and quality of life for older people (Estrada & Martínez, 2014).

Finally, with regard to satisfaction with one’s own life and subjective well-being, our findings through the articles included in the present review allow us to indicate the potential that exists from the direct relationship between both variables mentioned as central axes of the study (Dumitrache, Rubio & Cordon-Pozo, 2019; Huxhold, Fiori & Windsor, 2013). All this is supported by other studies mentioned throughout the text that allow us to affirm that there is a significant and therefore primordial relationship in healthy older people or those without cognitive impairment (Abello et al., 2008; Arita & Romano, 2005; Cuadra-Peralta et al., 2012; Diaz & Alvarado, 2007; Torres Palma & Flores Galaz, 2018).

### Study limitations

First, this systematic review has been influenced by the number of studies included and their heterogeneity. Therefore, the results should be viewed with caution. Also, the large variability in sample size and the wide range of ages that allow participants to be considered as older adults are one of the main sources of heterogeneity among the studies included in this review. Based on the current figures provided by the World Health Organization in the year 2021 (World Health Organization, 2021) in which it is estimated that the world population has a life expectancy of 60 years or more, and the number of relevant articles on the variables analyzed, the age of 60 years can be considered as a cut-off point for understanding the determinants of quality of life and well-being. Countries such as Peru consider older adults to be people who are 60 or older (Baldeón-Martínez et al., 2019) with a sample of 4,917 participants or in Spain (Pérez & Menor, 2015) with populations of healthy older adults aged 60 or older with a sample of 164 participants.

Second, there may have been a bias in the selection of the included papers.

Third, only articles published in serialized journals were included, so unpublished articles or searches in the “gray literature” were not taken into account.

Fourthly, the scarce scientific evidence on emotional intelligence and the factors that influence it has recommended that we include articles related to the emotional aspects that influence the quality of life of the cognitively healthy elderly person. These limitations give us a point to improve for future research.

Last but not least, on the one hand, future research can include in the study people with cognitive impairment or dementia that will broaden this research by providing new horizons. Therefore, it will allow us to be able to conduct research on the differences in emotional intelligence between elderly people with cognitive problems (dementia or cognitive impairment) and older adults who have a good cognitive status. At this point, it should be noted that it has not been possible to determine a concrete figure for the percentage of people aged 60 years or older who do not have any degree of cognitive impairment due to the lack of scientific evidence in this regard. However, there are scientific studies that can provide guidance on the above question, as they indicate the prevalence of Mild Cognitive Impairment (MCI), with a wide range worldwide. Thus, the results of different studies show a prevalence of 10% in individuals aged 70 to 79 years and 25% in people aged 80 to 89 years (Custodio et al., 2012). Another study in African Americans estimates prevalence rates of 19.2% for the age group 65 to 74 years, 27.6% for those aged 75 to 84 years and 38% for those aged 85 years and older (Parks et al., 2010). Thus, the prevalence of MCI is said to increase with age (Custodio et al., 2012; Parks et al., 2010). On the other hand, we were able to study the evolution of the factors that influence emotional intelligence, highlighting their relevance according to the different life stages, using samples of all ages and not only older people. This has been a limitation of our study in which only the elderly stage has been taken into account.

## Conclusions

Factors influencing emotional regulation facilitate older people’s adaptation to the changes that occur during ageing.

In addition, social support and personality have a direct relationship with life satisfaction and proactive coping strategies, showing a positive relationship with subjective well-being, life satisfaction and quality of life.

One of the strengths that can be found through the present review is the clinical usefulness of the results, since thanks to the knowledge of the psychological variables as the main determinants that influence the quality of life and well-being of older people without cognitive impairment, intervention programmes related to emotional education can be designed. The use of this type of programme can serve as a tool for the prevention of those illnesses that are currently so common in older adults, such as depression and anxiety, producing an improvement in the well-being and quality of life of these people.

Therefore, it can be concluded that social support and proactive coping strategies (emotional regulation strategies) are factors that influence emotional intelligence and have a positive effect on the well-being and quality of life of cognitively healthy older people.

## Supplemental Information

10.7717/peerj.12900/supp-1 Supplemental Information 1 Checklist.

## Additional Information and Declarations

### Data Availability

The following information was supplied regarding data availability: The raw measurements are available in the Supplemental Files.
